# A Dual Role for Death Receptor 5 in Regulating Cardiac Fibroblast Function

**DOI:** 10.3389/fcvm.2021.699102

**Published:** 2021-08-30

**Authors:** Miles A. Tanner, Laurel A. Grisanti

**Affiliations:** Department of Biomedical Sciences, College of Veterinary Medicine, University of Missouri, Columbia, MO, United States

**Keywords:** fibroblast, fibrosis, proliferation, apoptosis, ERK1/2 (p44/p42 MAPK), death receptor 5

## Abstract

The fibrotic response is involved in nearly all forms of heart failure and dysregulated responses can lead to enhanced cardiac dysfunction. TNF-related apoptosis-inducing ligand (TRAIL) and its receptor, death receptor (DR) 5, are associated with multiple forms of heart failure, but their role in the heart is poorly defined. Our previous study identified DR5 expression on cardiac fibroblasts however, the impact of DR5 on fibroblast function remains unexplored. To investigate the role of DR5 in cardiac fibroblasts, a variety of fibroblast functions were examined following treatment with the endogenous ligand, TRAIL, or small molecule agonist, bioymifi. DR5 activation did not induce apoptosis in naïve fibroblasts but activated ERK1/2 signaling to increase proliferation. However, upon activation and differentiation to myofibroblasts, DR5 expression was elevated, and DR5 agonists induced caspase 3 activation resulting in myofibroblast apoptosis. To investigate the impact of DR5 regulation of fibroblasts *in vivo*, a chronic isoproterenol administration model of heart failure was used. Wild-type (WT) mice receiving isoproterenol had increased hypertrophy, cardiomyocyte death, and fibrosis and decreased contractility compared to vehicle treated animals. DR5 knockout (KO) mice had no overt baseline phenotype however, following isoproterenol infusion, increased cardiomyocyte death and hypertrophy in comparison to isoproterenol treated WT animals was observed. DR5KO mice had an augmented fibrotic response with isoproterenol treatment compared with WT, which corresponded with additional decreases in contractility. These findings identify a dual role for DR5 in cardiac fibroblast function through enhanced naïve fibroblast proliferation, which switches to a pro-apoptotic function upon differentiation to myofibroblasts. This is important in heart failure where DR5 activation suppresses maladaptive remodeling and may represent a novel therapeutic target for the treatment of heart failure.

## Introduction

Cardiac fibroblasts play an important role in the normal and pathological heart through their ability to respond to stress or injury and facilitate wound healing responses (1). This response contributes to nearly all forms of heart disease, including those arising from ischemic and non-ischemic etiologies, and represents an important protective and reparative process. However, the fibrotic response can also have detrimental consequences when it is excessive through decreased cardiac compliance and furthering disease progression.

In the normal state, resident fibroblasts present in the heart contribute to homeostasis through the regulation of the extracellular matrix, which serves as a structural scaffold for the heart. Following stress or injury, resident fibroblasts become activated by various stimuli, including transforming growth factor (TGF)-β, proliferate (2–4), and transdifferentiate into myofibroblasts (5), which secrete large amounts of extracellular matrix and have contractile properties to repair the damaged tissue. Persistent myofibroblast activity has detrimental effects by augmenting cardiomyocyte death, increasing hypertrophy, and contributing to excessive extracellular matrix deposition. This decreases cardiac compliance and further progresses the disease. While targeting fibrosis has been an attractive therapeutic strategy for cardiac diseases, to date, clinical trials targeting fibrosis for the treatment of heart failure have been largely disappointing (6).

Recently, death receptor (DR; also called TRAIL-R2, TNFRSF10B) 5 and its ligand, TNF-Related Apoptosis-Inducing Ligand (TRAIL; also called Tnfsf10, APO2L, and CD253), have been linked to multiple forms of human heart failure, although the function of DR5 in the heart is poorly understood (7–9). In humans, TRAIL binds to two death-inducing receptors, DR4 (also called TRAIL-R1 and TNFRSF10A) and DR5 along with a receptor lacking the cytosolic domain, DcR1 (also called TNFRSF10C), and a receptor lacking the death domain, DcR2 (also called TNFRSF10D), which are thought to act as decoy receptors. TRAIL, DR4, DR5, DcR1, and DcR2 are highly expressed in the human heart at the transcript and protein level (10–14). Rodents express a single TRAIL death domain receptor, DR5, that is highly expressed in the heart (15) and homologous to human DR4 and DR5 (16). DR5 knockout (KO) mice have no overt baseline phenotype besides a reduction in thymus size (17). Canonical DR5 signaling leads to activation of the extrinsic pathway of apoptosis and has been an attractive therapeutic target for the treatment of cancer due to its ability to selectively induce apoptosis in transformed cells, whereas other cells tend to be resistant to TRAIL-induced apoptosis (18). Indeed, Phase I clinical trials have demonstrated no adverse effects of DR5 agonist administration (19–22). However, DR5 is ubiquitously expressed. Cardiomyocytes are capable of secreting TRAIL and recent studies from our laboratory demonstrated DR5 expression in cardiac fibroblasts, albeit at lower levels than cardiomyocytes, suggesting a role for localized TRAIL/DR5 signaling (23, 24). Interestingly, activation of DR5 in cardiomyocytes resulted in protective signaling suggesting that therapeutically targeting DR5 might be beneficial for the treatment of heart failure, which is in accordance with many of the clinical studies that suggest a beneficial role of TRAIL/DR5 (7–9). Furthermore, mice administered DR5 agonists do not have signs of increased cardiac fibrosis suggesting that DR5 agonists might be beneficial through activation of pro-survival signaling in cardiomyocytes and preventing excessive fibrosis (24). However, the impact of DR5 activation in cardiac fibroblasts has not been investigated.

The purpose of this study was to investigate the impact of DR5 activation on cardiac fibroblast function. Using an *in vitro* approach, activation of DR5 using the endogenous ligand, TRAIL, or small molecule agonist, bioymifi, did not activate apoptotic signaling or result in fibroblast death. In contrast, the proliferation of fibroblasts was enhanced, which occurred through ERK1/2 activation. However, upon differentiation to myofibroblast, the role of DR5 changed from pro-growth to pro-apoptotic. DR5 expression was increased in myofibroblasts and activation resulted in caspase 3 induction and apoptosis. These findings were confirmed *in vivo* using a chronic isoproterenol infusion model. DR5KO mice had enhanced fibrosis following isoproterenol administration compared with wild-type (WT) animals, despite similar levels of cardiomyocyte death and hypertrophy. This enhanced fibrosis correlated with a greater decline in contractility with isoproterenol. Taken together, this study identifies a role for DR5 in cardiac fibroblast where it enhances early fibrotic responses but dampens later fibrosis. These findings are important in heart failure where DR5 expression on fibroblasts plays an important role in diminishing adverse remodeling and may be a novel therapeutic target for the treatment of heart failure.

## Materials and Methods

### Experimental Animals

WT C57BL/6 mice or DR5KO mice (equal numbers male and female; 8–12 weeks old) were used in these studies. Mice were randomly assigned to groups and administered vehicle (saline+0.001% ascorbic acid) or isoproterenol (30 mg/kg/d) via osmotic minipump (Alzet). Mice were euthanized after 1-week administration and hearts were excised for experimental analysis. All animal procedures were performed with approval by the Institutional Animal Care and Use Committee at the University of Missouri and in accordance with the National Institutes of Health's *Guidelines on the Use of Laboratory Animals*.

### Echocardiography

Cardiac function was assessed via transthoracic 2D echocardiography performed using a VisualSonics Vevo 2100 System at baseline and weekly intervals following treatment using a 12-mHz probe on mice anesthetized with isoflurane (1.5%) as previously described (25). M-mode echocardiography was performed in the parasternal short-axis view at the level of the papillary to assess several cardiac parameters including left ventricular (LV) end-diastolic dimension, wall thickness, and LV fractional shortening.

### Neonatal Rat Ventricular Fibroblast Isolations

Primary neonatal cell cultures were prepared from 1 to 2 days old Sprague Dawley rat pups (Charles River) by enzymatic digestion as previously described (25). In brief, hearts were excised, cleaned, and ventricles digested using collagenase II (Worthington) and pancreatin. Myocytes were separated via pre-plating for 2 h. At isolation, adherent cells (fibroblasts) were at ~30% confluence. Following isolation, neonatal rat ventricular fibroblasts (NRVFs) were cultured in DMEM, 10% fetal bovine serum and 1% penicillin-streptomycin at 37°C in a humidified incubator with 5% CO_2_. Cells were passaged three times before use in experiments to eliminate contaminating cardiomyocytes. Dose-response curves were performed with increasing doses of TRAIL or bioymifi (Supplementary Figures 1A,B) and 100 pg/mL TRAIL and 1 μM bioymifi were chosen for treatment doses in subsequent experiments. ERK1/2 activation was examined following 30 min TRAIL or bioymifi treatment based on preliminary time course studies (Supplementary Figures 1C,D). Cell death and proliferation experiments were performed after 24 h treatment. To inhibit ERK1/2, cells received a 10 min pretreatment with 10 μM of the MEK1 inhibitor, PD98059 (Tocris, catalog # 1213). For differentiation to myofibroblasts, NRVFs were treated for 48 h with 5 ng/mL TGF-β1 (R&D Systems, catalog # 7666-MB-005).

### Adult Mouse Cardiac Fibroblast Isolations

Adult mouse cardiac fibroblasts (AMCFs) were isolated from WT C57BL/6 and DR5KO mice (male and female; 8–12 weeks old) as previously described (26, 27). Mice were euthanized and hearts were excised and flushed with Hank's Balanced Salt Solution to remove blood. Atria were removed and ventricles were digested by manual digestion into ~1 mm^3^ pieces and serial enzymatic digestion with collagenase II (150 U/mL) and trypsin (0.6 mg/mL) as previously described (28). Digestion steps were carried out in a shaking 37°C water bath. The collagenase II/trypsin solution was replaced after each digestion and the cell-containing solution was placed in a 50 mL Falcon tube with 4 mL fetal bovine serum. Subsequent digestions were carried out until the remaining tissue pieces were too small to separate from the digestion fluid (4–6 times). Myocytes were separated from fibroblasts by centrifugation at a low speed (845 × g for 5 min) and collecting the supernatant containing fibroblasts. Cells were cultured on 2% gelatin-coated plates in DMEM containing 10% fetal bovine serum and 1% penicillin-streptomycin at 37°C in a humidified incubator with 5% CO_2_ for 1 h prior to a media change to remove loosely adherent cells including myocytes and endothelial cells. To prevent phenotypic changes, AMCFs were not passaged, but immediately plated for use. For differentiation to myofibroblasts, AMCFs were treated for 48 h with 5 ng/mL TGF-β1 (R&D Systems, catalog # 7666-MB-005). Cells were plated one heart per 10 cm^2^ for ~30% confluence and received media changes every other day. Treatments occurred when AMCF were ~80% except for TGF-β1 treatments, which happened when cells were ~70% confluent so final treatments could be performed in differentiated cells that were ~80% confluent.

### Immunoblotting

NRVF samples were homogenized and resolved for immunoblot using standard protocols as previously described (23). Immunoblotting was performed with diluted antibodies against phospho-ERK1/2 (1:1,000; Cell Signaling, catalog # 9101), total-ERK1/2 (1:1,000; Cell Signaling, catalog # 4696), DR5 (1:1,000; R&D Systems, catalog # AF721), or GAPDH (1:1,000; Cell Signaling, catalog # 2118). After washing, membranes were incubated with the appropriate diluted secondary antibody and bound antibody was detected using the Azure Imaging System. Phosphorylated protein intensities were normalized to corresponding total protein intensities while other proteins were normalized to GAPDH.

### Reverse Transcription-Quantitative PCR

cDNA was synthesized from the total RNA of NRVF samples using the High-Capacity cDNA Reverse Transcription Kit (Applied Biosystems). RT-qPCR was performed with PowerUP SYBR Master Mix (Applied Biosystems) in triplicate for each sample using primers listed in Supplementary Table 1 at an annealing temperature of 60.1°C. All RT-qPCR data were analyzed using the Applied Biosystems Comparative CT Method (ΔΔCT). Gene expression analysis was normalized to translationally controlled tumor protein 1 (TPT1) and expressed as 2^−Δ*ΔCT*^ with min/max indicated for range.

### Immunofluorescence

Excised hearts were fixed in 4% paraformaldehyde, paraffin-embedded and sectioned at 5-μm thickness. Cardiomyocyte area was measured using fluorescein conjugated wheat germ agglutinin (WGA; 20 μg/ mL; Sigma-Aldrich) staining. Sections were visualized at 20X magnification using a Nikon Eclipse microscope and cell area was quantified in ImageJ (National Institutes of Health) from 10 fields/heart.

An *in situ* Cell Death Detection Kit, TMR Red (Roche Diagnostics) was used to measure apoptosis via terminal deoxynucleotidyl-transferase-mediated dUTP nick-end labeling (TUNEL) as previously described (25). DNA strand breaks were labeled according to the manufacturer's instructions using tetra-methyl-rhodamine-dUTP. Coverslips were counterstained with troponin I (1:100; Cell Signaling, catalog # 4002) to identify cardiomyocytes. Coverslips were mounted on glass slides using Prolong® Gold Antifade Reagent (Invitrogen). Slides were visualized at 20X magnification using a Nikon Eclipse microscope and the percentage of TUNEL-positive nuclei for random 10 fields were calculated in relation to the number of DAPI-stained nuclei.

Bromodeoxyuridine (BrdU) staining was performed on NRVFs as previously described (26). In brief, cells were incubated with BrdU labeling solution for 2 h at 37°C. Cells were rinsed and fixed with 4% paraformaldehyde followed by permeabilization with 0.2% Triton X-100 and treatment with 2M hydrochloric acid. Non-specific interactions were blocked with 0.1% bovine serum albumin and anti-BrdU antibody (1:100; R&D Systems, catalog # MAB7225) was incubated with cells overnight at 4°C followed by the appropriate Alexa488 conjugated secondary antibody. Nuclei were counterstained with DAPI and coverslips were mounted on glass slides using Prolong Gold Anti-Fade (Molecular Probes). Coverslips were visualized at 20X magnification using a Nikon Eclipse microscope from 10 randomly chosen fields and the percentage of positive BrdU nuclei for 10 fields/coverslip were calculated in relation to the number of DAPI-stained nuclei.

### Masson's Trichrome Staining

Masson's trichrome was performed as previously described and according to the manufacturer's instructions (Fisher Scientific) (29).

### Migration Assay

RNCFs were plated on 2% gelatin-coated plates and allowed to adhere for 24 h. Cells were serum starved for 1 h followed by scraping the cells in a straight line using a P200 pipette tip to create a scratch. Cells were stained with fluorescein WGA (20 μg/mL; Sigma-Aldrich) to outline the cell and imaged over time on a Nikon Eclipse microscope at 10X magnification. The rate of closer was calculated by normalizing the area of the scratch to the original area using ImageJ (National Institutes of Health, Bethesda, MD, US).

### Cell Viability Assay

NRVF viability was assessed using a CellTiter 96® Non-Radioactive Cell Proliferation (MTT) Assay (Promega). NRVFs were plated 20,000/well in a 96-well-plate. For NRVF survival experiments, cells were serum starved for 1 h prior to 24 h treatment with TRAIL (100 pg/mL) or bioymifi (1 μM). For proliferation experiments, media was replaced with fresh complete media 1 h prior to 24 h TRAIL or bioymifi treatment. Following treatment, Dye Solution was added to the plate followed by the Solubilization/Stop Solution after a 1 h incubation at 37°C. Absorbance was measure at 570 nm with a 750 nm reference wavelength.

### Caspase 3/7 Activity Assay

Caspase 3/7 activity was measured using a Caspase-Glo® 3/7 Assay according to the manufacturer's instructions (Promega). In brief, NRVMs were plated 20,000/well of a white-walled 96-well-plate. Cells were serum starved 1 h prior to TRAIL (100 pg/mL for 24 h) or bioymifi (1 μM for 24 h) treatment. After treatment, 100 μL of Caspase-Glo® 3/7 Reagent was added to each well. Plates were incubated for 1 h prior to reading.

### Lactate Dehydrogenase Assay

NRVF cell death was measured using a Lactate Dehydrogenase (LDH) Colorimetric Assay Kit according to the manufacturer's instructions (Pierce). Release of LDH was determined in media from NRVMs treated with TRAIL (100 pg/mL for 24 h) or bioymifi (1 μM for 24 h). Absorbance was measured by a spectrophotometer at 490 and 680 nm. LDH activity was expressed as fold of vehicle.

### Statistical Analysis

Data presented are expressed as mean ± SEM. Statistical analysis was performed using unpaired Student *t*-test, one-way ANOVA with a Tukey's multiple comparison test, or two-way repeated-measures ANOVA where appropriate using Prism 5.0 software (GraphPad Software), with *p*-values indicated in the figure legends.

## Results

**Figure 1:**
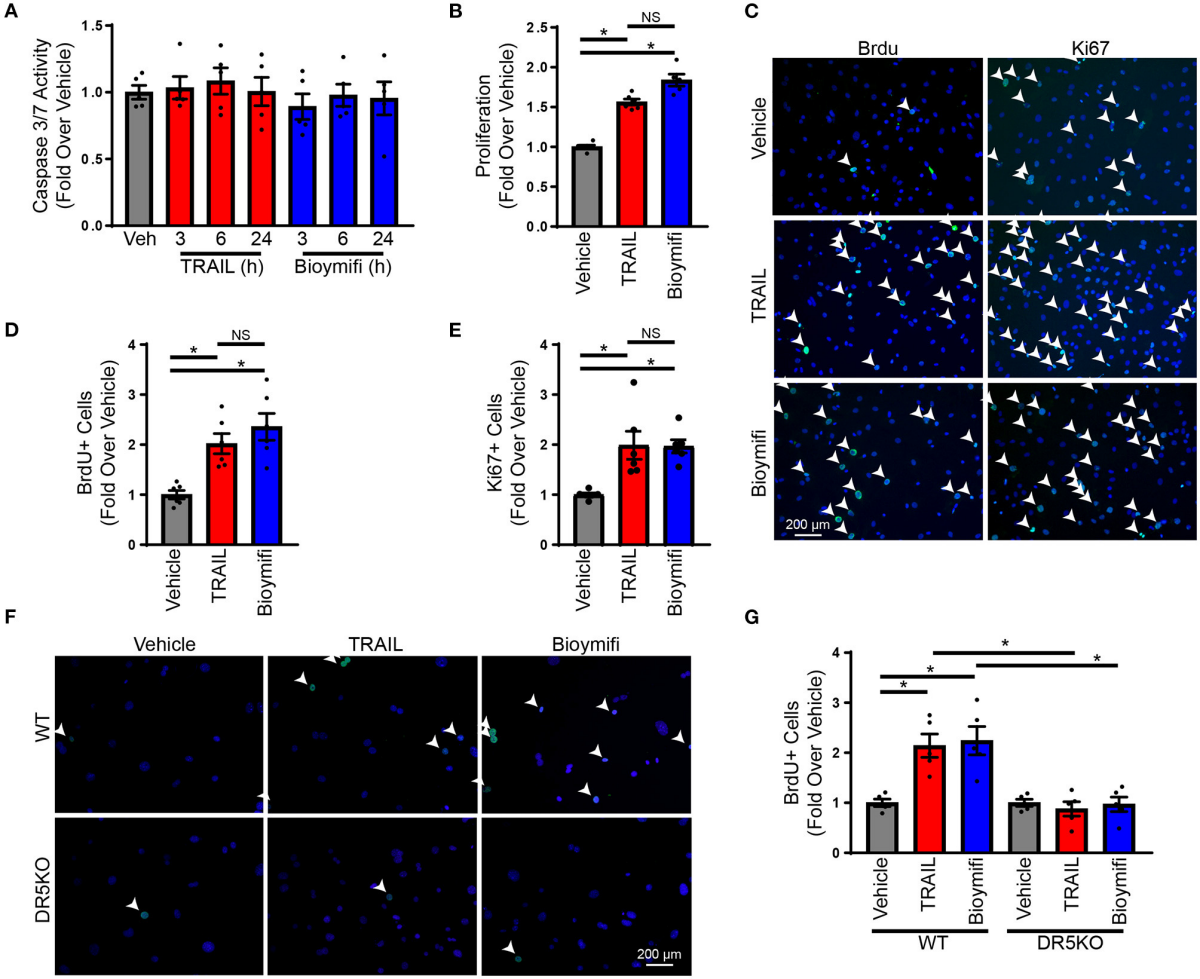
DR5 activation induces proliferation in cardiac fibroblasts. **(A)** A caspase 3/7 assay was used to measure the induction of apoptosis in NRVFs treated over time with TRAIL, bioymifi, or vehicle control. *N* = 5. **(B)** An MTS assay was used to measure proliferation in vehicle, TRAIL, or bioymifi treated NRVFs. *n* = 6, One-Way ANOVA, *p < 0.05. **(C)** Representative BrdU and Ki67 staining (green) and quantified BrdU **(D)** or Ki67 **(E)** staining for vehicle, TRAIL, or bioymifi treated NRVFs. Cells were counterstained with DAPI (blue) to identify nuclei. White arrows indicate BrdU-positive nuclei. *n* = 6, One-Way ANOVA, *p < 0.05. Representative **(F)** and quantified **(G)** BrdU staining (green) in WT and DR5KO AMCF treated with TRAIL, bioymifi, or vehicle control. DAPI (blue) was used to identify nuclei. White arrows indicate BrdU-positive nuclei. *n* = 5, Two-Way ANOVA, *p < 0.05.

Our previous studies identified expression of DR5 on cardiac fibroblasts; however, at lower levels than cardiomyocytes (24). This was confirmed in neonatal rat ventricular myocytes (NRVMs) and NRVFs (Supplementary Figure 2), which identified roughly 50% of the expression of DR5 in NRVFs compared with NRVMs. To determine if DR5 activation results in cardiac fibroblast apoptosis, NRVFs were treated over time with the endogenous DR5 agonist, TRAIL, or the small molecule DR5 agonist, bioymifi, and apoptosis was examined using a caspase 3/7 activity assay. Caspase 3/7 activity was unaltered with DR5 activation in response to TRAIL or bioymifi (Figure 1A). Furthermore, no alterations in cell death (Supplementary Figure 3A) or survival (Supplementary Figure 3B) were observed demonstrating that DR5 does not induce death in NRVFs.

To determine the role of DR5 in cardiac fibroblasts, the influence of DR5 activation on a variety of fibroblast functions was examined including cytokine production, migration, myofibroblast differentiation, and proliferation. Treatment with TRAIL or bioymifi had no impact on TNFA (Supplementary Figure 4A) or IL6 expression (Supplementary Figure 4B), migration (Supplementary Figures 4C,D) or myofibroblast differentiation (Supplementary Figures 4E,F). Proliferation of NRVFs was enhanced with TRAIL and bioymifi treatment when examined for metabolic activity using an MTS assay (Figure 1B), DNA replication by BrdU staining (Figures 1C,D; Supplementary Figure 5A) or mitosis using Ki67 staining (Figures 1C,E). To confirm the importance of DR5 activation in the proliferative response to TRAIL and bioymifi, AMCF were isolated from WT and DR5KO animals and treated with TRAIL, bioymifi, or vehicle control. Similar to NRVFs, TRAIL, and bioymifi increased BrdU incorporation in WT adult cardiac fibroblasts, which did not occur in DR5KO AMCF (Figures 1F,G; Supplementary Figure 5B). These data suggest TRAIL and bioymifi enhance fibroblast proliferation through DR5-mediated mechanisms.

**Figure 2:**
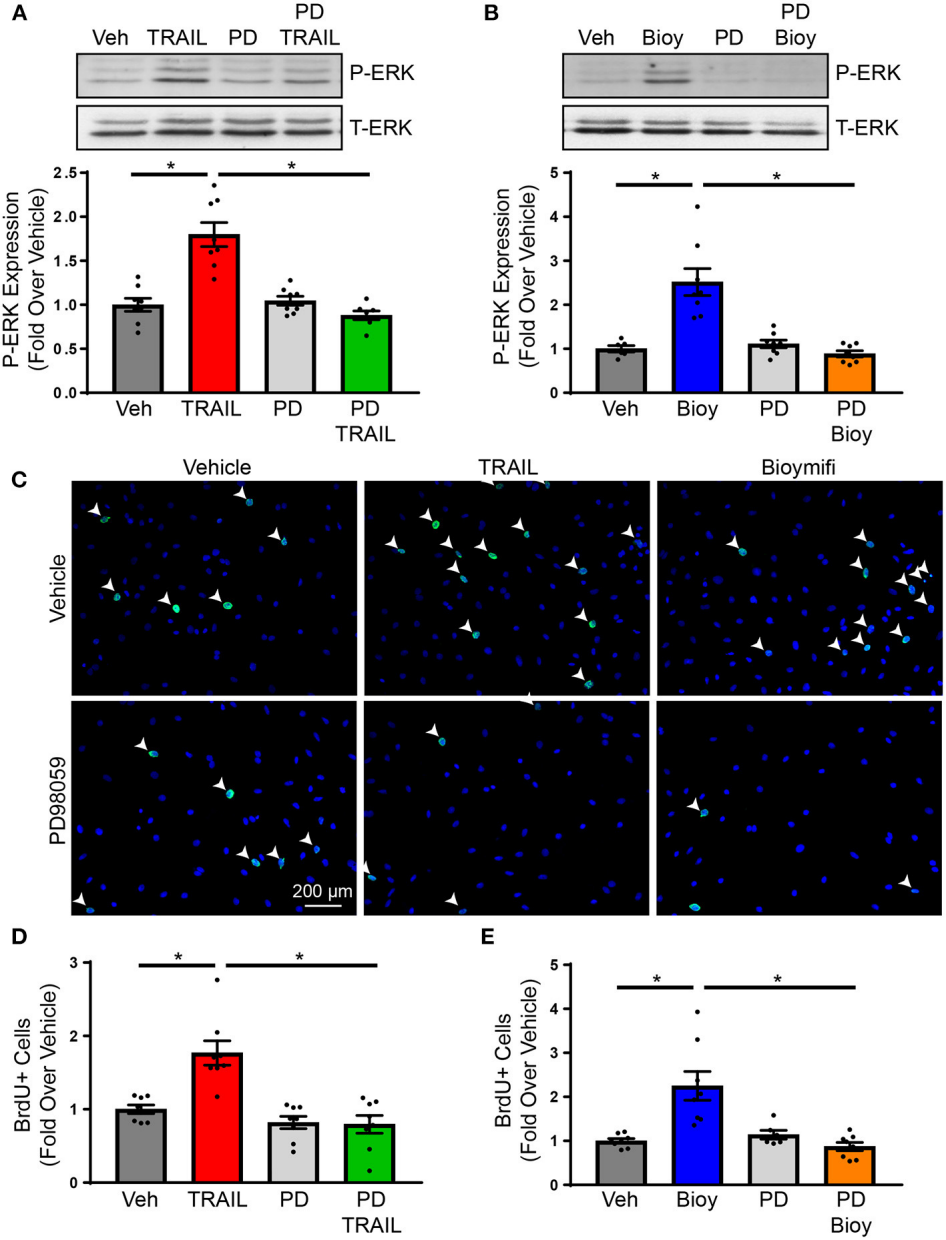
DR5-mediated changes in proliferation occur through ERK1/2 activation. **(A)** ERK1/2 activation in response to TRAIL in NRVFs was examined using immunoblot analysis for phospho-ERK1/2 in the presence or absence of PD98059. Total-ERK1/2 is shown as a loading control. *n* = 8, One-Way ANOVA, *p < 0.05. **(B)** Phosphorylation of ERK1/2 was examined in bioymifi treated NRVFs in the presence or absence of PD98059. Total-ERK1/2 is shown as a loading control. *n* = 8, One-Way ANOVA, *p < 0.05. **(C)** Representative BrdU staining (green) and quantified BrdU staining for TRAIL **(D)** or bioymifi **(E)** treated NRVFs with our without PD98059 pretreatment. DAPI staining (blue) is used to show nuclei. White arrows indicate BrdU-positive nuclei. *n* = 8, One-Way ANOVA, *p < 0.05.

Previous findings from our laboratory identified ERK1/2 phosphorylation in the hearts of mice treated with DR5 agonists (24). While this was attributed to ERK1/2 activation in cardiomyocytes, ERK1/2 is a pleiotropic kinase and is known to increase cardiac fibroblasts proliferation in response to various ligands. However, the influence of DR5 on ERK1/2 activation in cardiac fibroblast was not examined (30–32). To determine if ERK1/2 is phosphorylated in response to DR5 activation in cardiac fibroblast, NRVFs were treated over time with TRAIL or bioymifi and phospho-ERK1/2 was examined by immunoblot (Supplementary Figures 1C,D). ERK1/2 was phosphorylated with DR5 activation by TRAIL (Supplementary Figure 1C) or bioymifi (Supplementary Figure 1D) with levels peaking at 30 min. Activation of ERK1/2 by TRAIL (Figure 2A) or bioymifi (Figure 2B) was inhibited by pretreatment with the MEK1 inhibitor, PD98059, demonstrating the specificity of the response.

To determine if ERK1/2 activation contributes to the proliferative actions of DR5 agonists, NRVFs were treated with TRAIL or bioymifi in the presence or absence of PD98059. Activation of DR5 by TRAIL (Figures 2C,D; Supplementary Figure 5A) or bioymifi (Figures 2C,E; Supplementary Figure 6A) resulted in an increase in the number of BrdU-positive NRVFs. PD98059 alone had no impact on proliferation, whereas PD98059 in combination with TRAIL or bioymifi prevented DR5-mediated increases in BrdU staining (Figures 2C–E). These studies confirm the involvement of ERK1/2 in the proliferative actions of DR5 agonists. Since fibroblast proliferation is a primary contributor to fibrosis (1), this data would suggest increased cardiac fibrosis with DR5 activation *in vivo*. However, our previous study demonstrated *in vivo* activation of DR5 using small molecule or antibody agonist administration resulted in no change in fibrosis, which suggests DR5 may be acting through other mechanisms to suppress fibrosis (24).

**Figure 3:**
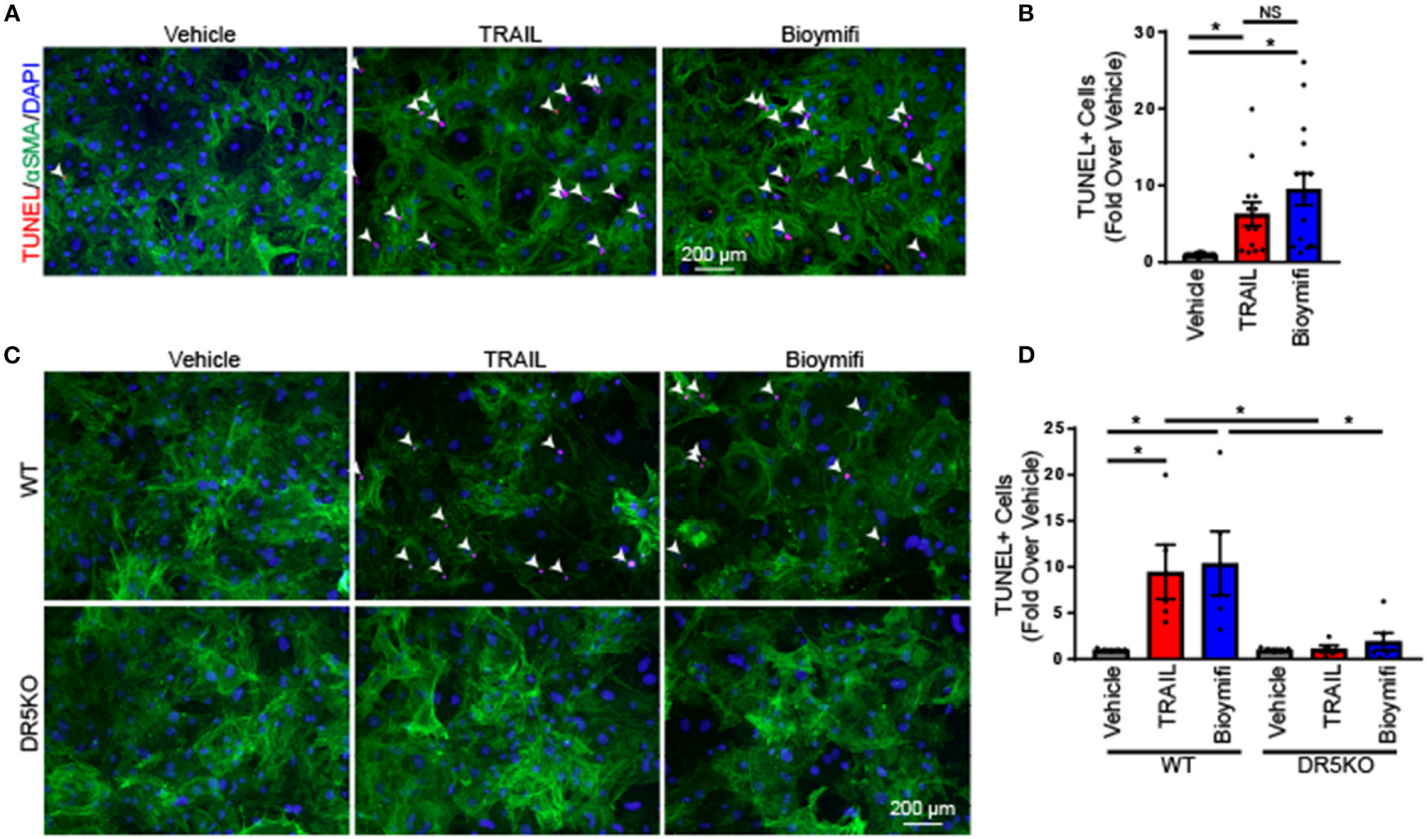
DR5 activation induced death in myofibroblasts. **(A)** Apoptosis was examined by TUNEL staining (red) in rat neonatal myofibroblasts treated with vehicle, TRAIL, or bioymifi. αSMA staining (green) was used to label myofibroblasts and DAPI staining (blue) shows nuclei. **(B)** Quantification of the number of TUNEL-positive nuclei in myofibroblasts treated with TRAIL, bioymifi, or vehicle control. *n* = 8. One-Way ANOVA, *p < 0.05. Representative **(C)** and quantified **(D)** TUNEL staining for myofibroblasts from adult WT and DR5KO mouse hearts treated with TRAIL or bioymifi. αSMA staining (green) was used to label myofibroblasts and DAPI staining (blue) shows nuclei. Data presented as fold over vehicle from the same mouse. *n* = 5, Two-Way ANOVA, *p < 0.05.

During pathological conditions, fibroblasts become activated and transdifferentiate to myofibroblasts, which hyper-secrete extracellular matrix and have contractile properties to facilitate wound repair (1). To determine the impact of DR5 activation in myofibroblasts, NRVFs were differentiated into myofibroblasts using TGF-β1 as indicated in the “Methods.” Myofibroblasts were treated with TRAIL or bioymifi and cell death was examined by TUNEL staining. In contrast with naïve fibroblasts, myofibroblasts had increased cell death following TRAIL or bioymifi treatment (Figures 3A,B) suggesting a change in DR5 function upon differentiation. Furthermore, WT and DR5KO myofibroblasts from adult mice also demonstrated TRAIL and bioymifi induced myofibroblast death in adult WT myofibroblasts and DR5 agonist-mediated death does not occur in DR5KO cells (Figures 3C,D). This shows that myofibroblast death in response to DR5 ligands is a receptor-specific response.

**Figure 4:**
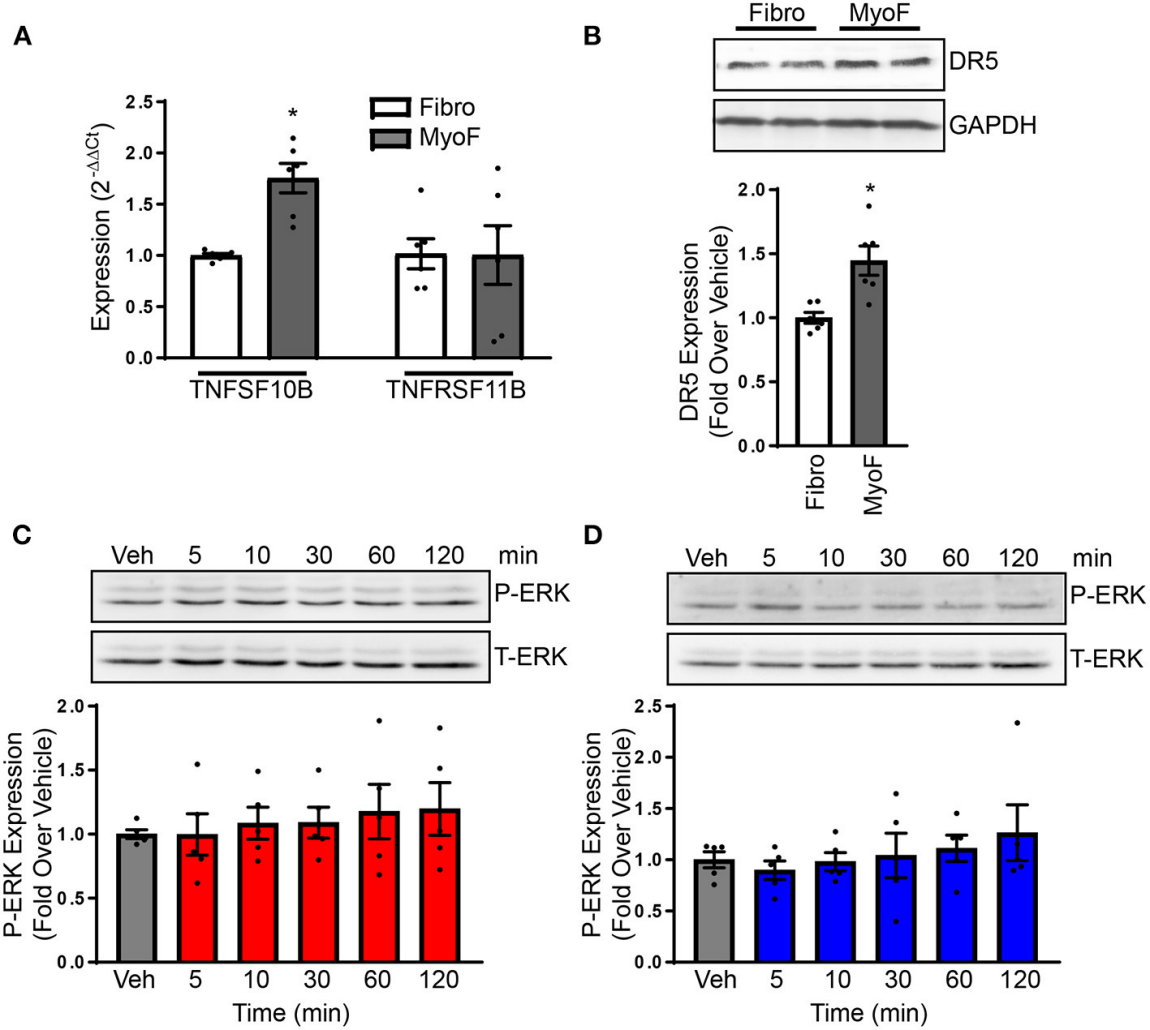
**(A)** DR5 and OPG transcript expression was examined with NRVFs and myofibroblasts by RT-qPCR and expressed relative to NRVF transcript levels. *n* = 6. *T*-test, *p < 0.05. **(B)** DR5 protein expression was detected via immunoblot in NRVFs and myofibroblasts. GAPDH is shown as a loading control. The molecular weight ladder (Ldr) is shown in the right lane. Values are expressed relative to NRVFs. *n* = 6. *T*-test, *p < 0.05. ERK1/2 activation was examined in myofibroblasts in response to TRAIL **(C)** or bioymifi **(D)** treatment over time by immunoblot for phospho-ERK1/2. Total-ERK1/2 is shown as a loading control. *n* = 5.

A major regulatory mechanism of TRAIL signaling is through alterations in its receptors. To identify if TRAIL receptors are altered between naïve fibroblast and myofibroblast, expression was examined. TNFRSF10B expression, the gene for DR5, was increased in myofibroblasts in comparison to naïve fibroblasts (Figure 4A), which also occurred at the protein level (Figure 4B). Expression of TNFRSF11B, the gene for osteoprotegerin (OPG), a TRAIL decoy receptor, was unaltered (Figure 4A). Unfortunately, rodent equivalents of DcR1 and DcR2 have not yet been identified so other decoy receptor expression could not be examined (16). To determine if ERK1/2 is activated in myofibroblasts, cells were treated over time with TRAIL or bioymifi, followed by immunoblotting for phosphorylated ERK1/2. In contrast with naïve NRVFs, myofibroblasts did not have increased ERK1/2 phosphorylation with TRAIL or bioymifi treatment at any time point examined (Figures 4C,D). These findings show that the signaling mechanisms of DR5 change once fibroblasts become activated.

**Figure 5:**
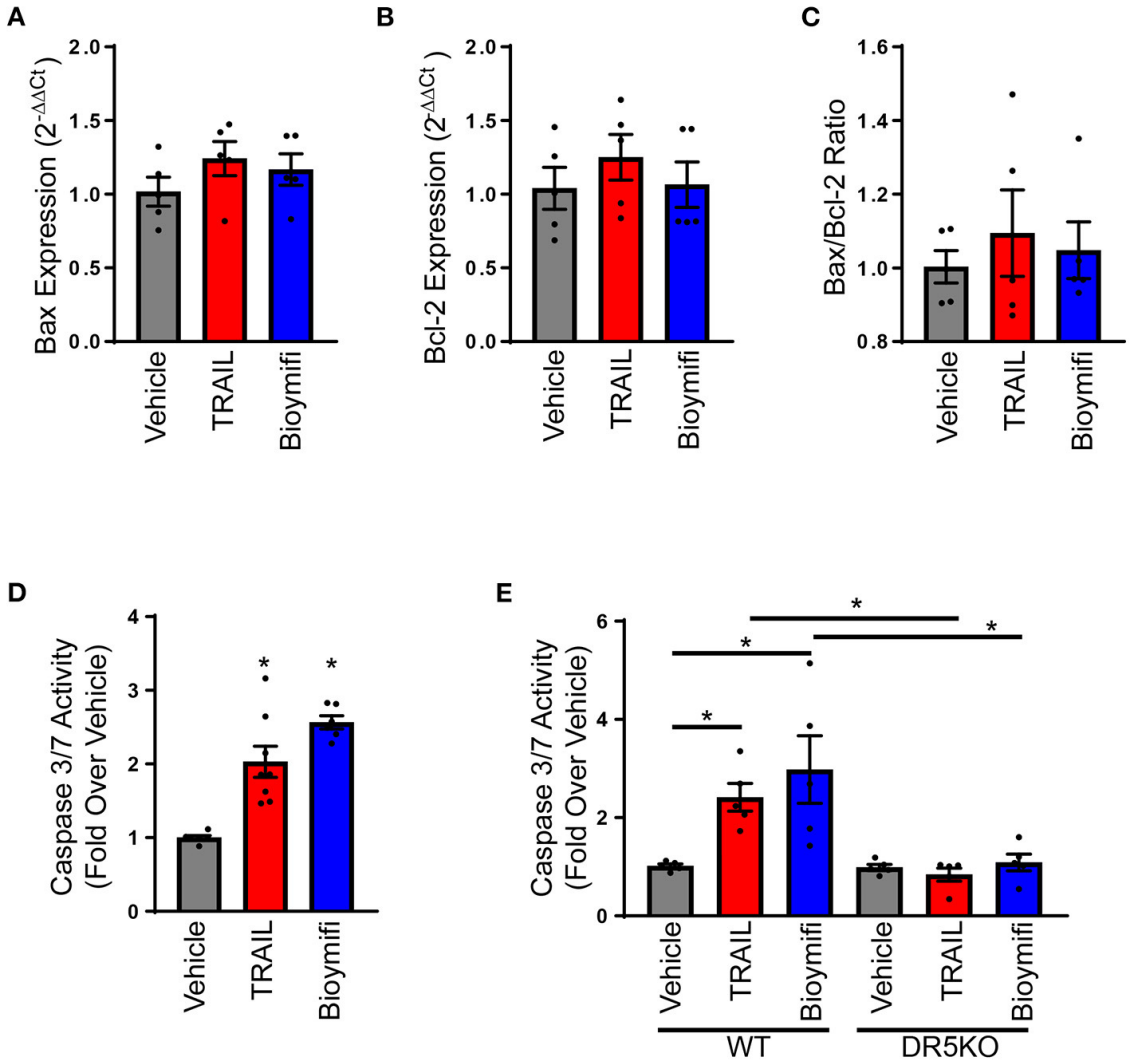
DR5 activation induces apoptosis in myofibroblasts. Bax **(A)** and Bcl-2 **(B)** transcript expression and the calculated Bax to Bcl-2 ratio **(C)** were examined in neonatal rat myofibroblasts treated with vehicle, TRAIL or bioymifi using RT-qPCR and expressed relative to vehicle treated cells. *n* = 5. **(D)** A Caspase 3/7 Activity assay was used to measure apoptosis in myofibroblasts treated with TRAIL, bioymifi, or vehicle control. Values are expressed relative to vehicle. *n* = 6, One-Way ANOVA, *p < 0.05 vs. vehicle. **(E)** Apoptosis was quantified in WT and DR5KO myofibroblasts from adult mouse heart and treated with vehicle, TRAIL or bioymifi. Values are expressed relative to WT vehicle. *n* = 5, One-Way ANOVA, *p < 0.05.

Since DR5 activation increases apoptosis in myofibroblasts and canonical DR5 signaling results in terminal caspase activation and cell apoptosis through mitochondria-dependent or independent mechanisms (33), we wanted to determine DR5 was increasing myofibroblast death by promoting apoptotic signaling. In cancer cells, DR5 is known to induce apoptosis through multiple pathways including mitochondria-dependent and independent mechanisms. To determine if the mitochondrial apoptotic pathway is activated in cardiac myofibroblasts with DR5 stimulation, myofibroblasts were treated with TRAIL or bioymifi and the pro-apoptotic factor, Bax, and anti-apoptotic factor, Bcl-2, were examined. No alterations were observed in the transcript expression of Bax (Figure 5A) or Bcl-2 (Figure 5B) with TRAIL or bioymifi. The ratio of Bax and Bcl-2, often considered the most accurate indicator of apoptosis susceptibility, was also unchanged with TRAIL or bioymifi treatment (Figure 5C). These findings suggest the mitochondrial apoptotic pathway is not activated by DR5 agonists in cardiac myofibroblasts.

To determine if caspase 3 was activated with DR5 agonists, myofibroblasts were treated with TRAIL or bioymifi over time and a caspase 3/7 activity assay was performed. Activation of DR5 by TRAIL or bioymifi resulted in increased caspase 3/7 activity, demonstrating that DR5 induces apoptosis in myofibroblasts (Figure 5D). Additionally, TRAIL and bioymifi activated caspase 3/7 in myofibroblasts derived from WT AMCF while having no impact on myofibroblasts from DR5KO cells (Figure 5E).

**Figure 6:**
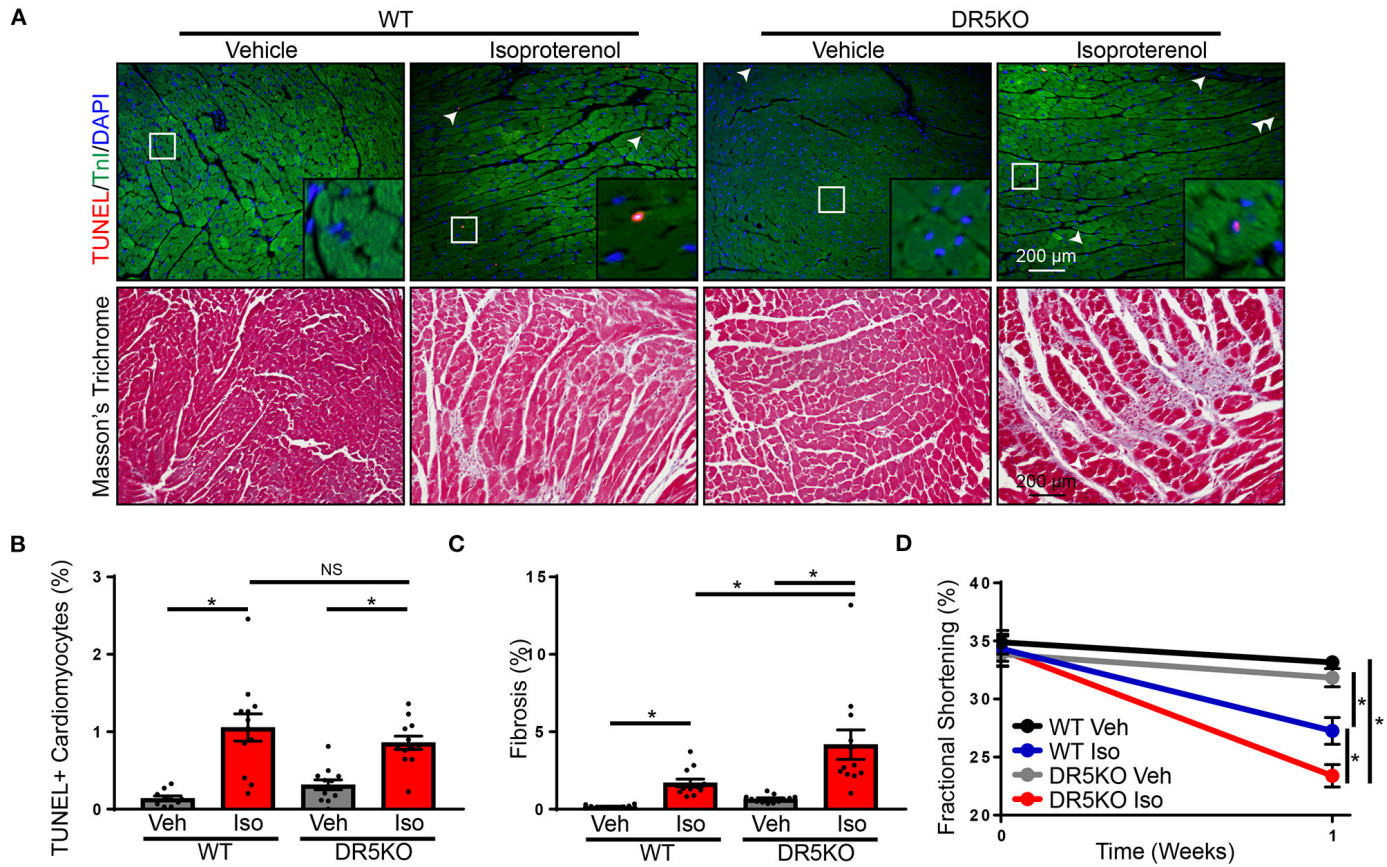
DR5KO exacerbates fibrosis *in vivo*. **(A)** *Top:* Representative TUNEL (red) staining in hearts of WT and DR5KO animals administered vehicle or isoproterenol. Cardiomyocytes were identified by troponin I (TnI) staining (green) and nuclei were labeled using DAPI (blue). TUNEL positive nuclei are indicated by white arrows. *Bottom:* Representative Masson's trichrome staining showing fibrosis (blue) in hearts from vehicle and isoproterenol treated WT and DR5KO mice. **(B)** Quantification of TUNEL positive cardiomyocytes from WT and DR5KO mice administered vehicle or isoproterenol. *n* = 10 for WT vehicle, 12 for DR5KO vehicle, WT isoproterenol, and DR5KO isoproterenol, Two-Way ANOVA, *p < 0.05 vs. vehicle. **(C)** Quantification of fibrotic area from Masson's trichrome stained WT and DR5KO hearts after vehicle or isoproterenol treatment. *n* = 10 for WT vehicle, 12 for DR5KO vehicle, WT isoproterenol and DR5KO isoproterenol, Two-Way ANOVA, *p < 0.05. **(D)** Fractional shortening was measured echocardiography in WT and DR5KO mice at baseline and with vehicle or isoproterenol infusion. *n* = 10 for WT vehicle, 12 for DR5KO vehicle, WT isoproterenol and DR5KO isoproterenol, Two-Way ANOVA, *p < 0.05.

Our *in vitro* findings suggest DR5 activation may be an anti-fibrotic mechanism by inducing apoptosis in activated fibroblasts. To examine the physiological impact of these findings *in vivo*, a chronic isoproterenol administration model of heart failure was used. WT and DR5KO mice were administered isoproterenol or vehicle control as outlined in the “Methods” portion of this manuscript. This model has many of the classic hallmarks of heart failure including increased cardiomyocyte death, hypertrophy, and fibrosis and decreased contractility. To examine the impact of DR5KO on cell death, a TUNEL assay was performed. Isoproterenol administration increased the number of TUNEL-positive cardiomyocytes in comparison with vehicle treated WT mice (Figures 6A,B). Vehicle treated DR5KO mice were not different from vehicle treated WT mice, which is unsurprising since DR5KO mice have no overt cardiac phenotype under normal conditions (17). Isoproterenol increased the number of TUNEL-positive cardiomyocytes in DR5KO mice, but levels were unaltered from isoproterenol treated WT animals. Similarly, hypertrophy at the organ and cellular level were increased in WT and DR5KO mice with isoproterenol infusion, but levels were unaltered between genotypes (Supplementary Figure 7, Supplementary Table 2.). Masson's trichrome staining was performed to determine levels of fibrosis in WT and DR5KO animals administered isoproterenol or vehicle control. As expected, isoproterenol increased the amount of fibrosis observed in WT mice compared with vehicle control treated animals (Figures 6A,C). DR5KO mice had an augmented fibrotic response following isoproterenol treatment compared with WT despite vehicle treated animals being similar to WT. Furthermore, fractional shortening, a measure of contractility, was impaired with isoproterenol infusion in WT mice, which was further decreased in DR5KO animals (Supplementary Figure 8, Figure 6D). These findings demonstrate that DR5 activation *in vivo* reduces fibrosis without altering cardiomyocyte death or hypertrophy and these changes improve diastolic dysfunction.

## Discussion

Fibroblasts play an important role in the normal heart through their contribution to the structural organization and homeostasis of the heart (1). They also play a critical role in response to injury and in the diseased heart where the fibrotic response contributes to nearly all diseases states. TRAIL and its receptor DR5 have been associated with multiple types of heart failure in multiple clinical and mouse models of heart failure (7, 8, 10, 34–37). Our laboratory has recently identified a role for DR5 activation in regulating pro-hypertrophic signaling in cardiomyocytes (24). In this study, DR5 expression was identified on cardiac fibroblasts however, its function was not investigated (24). The purpose of the current study was to characterize the impact of DR5 on cardiac fibroblast function. In naïve fibroblasts, DR5 agonists failed to activate canonical apoptotic signaling pathways, but increased fibroblast proliferation through the activation of ERK1/2. However, upon activation and differentiation into myofibroblasts, DR5 expression was upregulated and signaling was altered. In myofibroblasts, DR5 activation induced apoptosis leading to increased myofibroblast death. This impacted fibroblasts function *in vivo* where DR5KO augmented the fibrotic response in a chronic isoproterenol administration model of heart failure.

While our laboratory has previously shown DR5 expression on cardiac fibroblasts (24), the present study is the first to examine the impact of DR5 on cardiac fibroblast function. However, other groups have investigated DR5 in other fibroblast populations. Several fibroblast populations, including normal human lung fibroblasts and dermal fibroblasts, are resistant to TRAIL-induced apoptosis despite high expression of DR4 and DR5 (20, 38, 39). In a separate study examining a panel of human fibroblast cell lines from a variety of origins, non-malignant fibroblasts were found to be resistant to TRAIL-induced apoptosis whereas malignant cell lines were susceptible to TRAIL-mediated cell death (40). It is not clear what causes non-apoptosis-inducing TRAIL signaling in non-transformed cell types, but it is thought that upregulation of decoy receptors or alterations in the apoptotic machinery in these cell types could contribute to these changes (39). In certain fibroblast populations, changes in the apoptotic machinery appear to be responsible for TRAIL-resistance. Studies have demonstrated that TRAIL enhances caspase 8 degradation in TRAIL-sensitive fibroblasts in comparison with malignant, TRAIL-resistant fibroblasts (39, 40).

While canonical DR5 signaling occurs through typical death receptor signal transduction pathways culminating in the activation of caspases, several alternative signaling mechanisms for DR5 have been identified (41). The present study demonstrated ERK1/2 activation in naïve fibroblasts following TRAIL or bioymifi treatment and previous studies in mice using DR5 activating antibodies or bioymifi treatment have shown ERK1/2 activation in the heart (24). ERK1/2 has been implicated in proliferative and protective pathways in response to TRAIL in a number of different cell types. In small cell lung cancer cells lacking caspase 8, TRAIL induced proliferation, which could be inhibited using ERK1/2 siRNA (42). TRAIL-resistant human glioma cells and vascular endothelial cells have increased TRAIL-mediated proliferation that is prevented by pharmacological ERK1/2 inhibition (43, 44). ERK1/2 signaling is also known to induce proliferation in many cell types, including fibroblasts, in response to multiple ligands including growth factors and G protein-coupled receptors (26, 45). ERK1/2 is known to impact proliferation through the regulation of transcription factors including AP-1 and Elk-1 (46). Thus, it is unsurprising that ERK1/2 activation via DR5 induced proliferation in cardiac fibroblasts.

The change from a proliferative to a pro-apoptotic role for DR5 between naïve fibroblasts and myofibroblasts suggests different signal transduction paradigms between cell types. Indeed, while ERK1/2 was activated with DR5 agonists in naïve fibroblasts, no ERK1/2 response was observed in myofibroblasts. In contrast, caspase 3 activation was observed suggesting a switch to canonical death receptor signaling mechanisms. TRAIL and its receptors have been extensively studied in the cancer field due to their ability to induce apoptosis in transformed cell types (39). This is particularly evident in clinical trials using DR5 agonists where adverse effects were not observed (19). However, TRAIL-resistant cell types are common or can arise following therapy, which often occurred through a regulation of receptor levels (36, 39). Similar to this, we observed differences in DR5 expression following fibroblast activation, which suggests an imbalance of pro-apoptotic and decoy TRAIL receptors and resulted in increased TRAIL-induced apoptosis. This has also been observed in dermal fibroblasts where a DR5 agonist prevents human dermal fibroblasts' activation to myofibroblasts by inducing myofibroblast apoptosis (47). Similar to our study, dermal fibroblasts upregulated DR4 and DR5 expression during fibroblast activation, which occurred via Smad signaling. However, DR5 signaling may be regulated in different ways. Membrane localization has been shown to be important for directing signaling in some cell types and dysregulations in DR5 localization has been shown to contribute to TRAIL-resistance in cancer cells (48). Alterations in DR5 positioning at the membrane may alter adapter protein interactions culminating in differences in downstream signaling. ERK1/2 is a pleiotropic kinase and compartmentalization of signaling has also been shown to play an important role in directing ERK1/2 signaling to particular outcomes (49). Apoptotic signaling by TRAIL can occur through the formation of a DISC complex and direct activation of caspases or indirectly through inducing the transcription of mitochondrial apoptotic machinery (41). We did not see alterations in Bax or Bcl-2 despite increased caspase 3/7 activity with DR5 activation in myofibroblasts. This suggests direct caspase activation occurs with DR5 agonists upon activation of fibroblasts to myofibroblasts.

TRAIL and/or DR5 have been associated with multiple heart failure types (7, 8, 10, 34–37). Interestingly, many of these studies suggested a protective role for the TRAIL/DR5 system in heart failure (7–9, 35, 50). Additionally, OPG, which can act as a TRAIL decoy receptor, has been extensively studied in the setting of the heart. In heart failure, increased levels of OPG are associated with worsened prognoss (51–53). However, its impact on the TRAIL system is rarely considered and how OPG impacts DR5 signaling remains to be elucidated. In addition to clinical correlates, TRAIL and its receptor have been shown to be elevated in a mouse model of heart failure (23). Mechanically stretched cardiomyocytes, heart sections treated with isoproterenol and simulated ischemia/reperfusion injury in human atrial samples have been shown to release TRAIL, suggesting a role in localized signaling to the heart (35, 54, 55). To determine the impact of DR5 on fibrosis *in vivo*, a chronic isoproterenol administration of heart failure was used. WT and DR5KO mice had similar levels of hypertrophy and cardiomyocyte death with isoproterenol infusion suggesting that differences in fibrosis are a result of DR5 activation on fibroblasts rather than cardiomyocytes. However, there is extensive crosstalk between different populations of the heart and these studies and previous studies using DR5 agonist administration (24), are global in nature. Further studies using cell type-specific KO would be beneficial in fully elucidating the cell-specific roles of DR5 in the heart. DR5KO mice had an augmented fibrotic response with isoproterenol infusion. This suggests that DR5 is important for reducing the fibrotic response in the heart after injury and aligns with our *in vitro* findings showing DR5 activation induces apoptosis in myofibroblasts. In addition to increased fibrosis, DR5KO mice have impaired contractility following isoproterenol administration when compared with WT mice. This is unsurprising since fibrosis is known to impair mechano-electric coupling of cardiomyocytes and increase myocardial stiffness (56).

**Figure 7:**
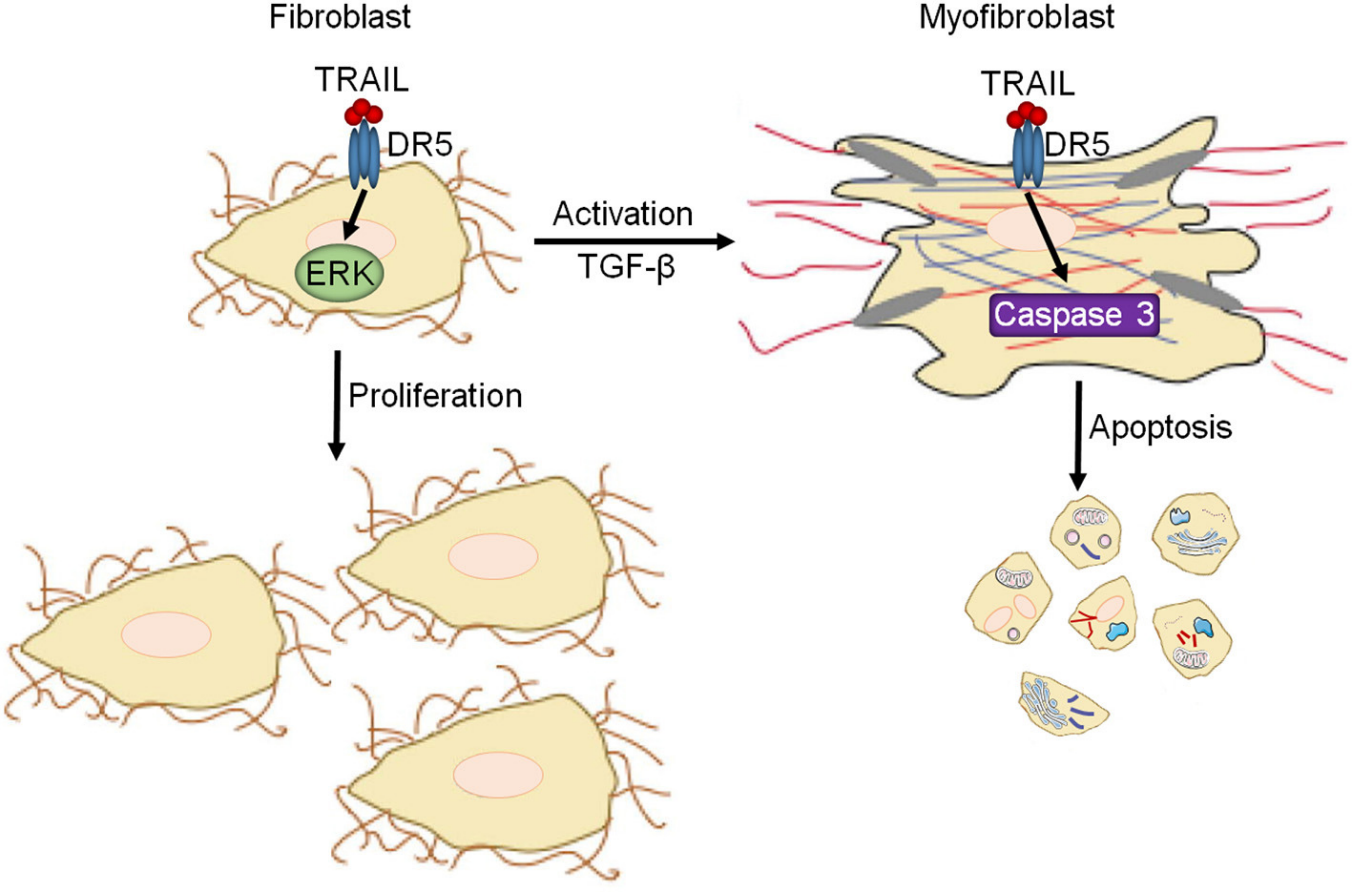
Summary of DR5 in cardiac fibroblasts. In naïve cardiac fibroblasts, DR5 activates ERK1/2 resulting in increased proliferation. Upon differentiation/activation of cardiac fibroblasts to myofibroblasts, DR5 signaling changes to a canonical DR5 signaling paradigm leading to caspase activation and cell death.

Heart failure is a significant healthcare problem and a leading cause of death. Despite its significance, few advances have been made recently to improve outcome for heart failure patients and current therapies rely on short-term improvements in clinical status and do little for long-term prognosis. This demonstratthese a need for novel therapeutic targets and treatment strategies. Fibrosis is an attractive therapeutic target for the treatment of heart failure due to its contribution to multiple heart failure etiologies where is starts as an adaptive repair mechanism, but ultimately potentiates disease progression through myocardial stiffening (1). However, to date, no anti-fibrotic therapies have successfully translated to clinical trials demonstrating the need for novel approaches. Our previous study demonstrated that DR5 leads to protective signaling in cardiomyocytes (24). The current study shows that DR5 has the ability to potentiate early fibrotic responses through its role in increasing naïve fibroblast proliferation via ERK1/2 signaling. However, upon fibroblast activation, the function and signaling of DR5 switches to a pro-apoptotic role through the suppression of ERK1/2 signaling and activation of terminal caspases, thus limiting fibrosis (Figure 7). This modulatory role for DR5 in fibroblast function shows that therapeutically targeting DR5 in the heart may offer protection in heart failure and provide a novel, beneficial strategy for the treatment of heart failure.

## Data Availability Statement

The original contributions presented in the study are included in the article/Supplementary Material, further inquiries can be directed to the corresponding author/s.

## Ethics Statement

The animal study was reviewed and approved by Institutional Animal Care and Use Committee at the University of Missouri.

## Author Contributions

MT and LG performed the experiments. LG conceptualized, designed the experiments, analyzed the data, and wrote the manuscript. All authors have read and agreed to the published version of the manuscript.

## Conflict of Interest

The authors declare that the research was conducted in the absence of any commercial or financial relationships that could be construed as a potential conflict of interest.

## Publisher's Note

All claims expressed in this article are solely those of the authors and do not necessarily represent those of their affiliated organizations, or those of the publisher, the editors and the reviewers. Any product that may be evaluated in this article, or claim that may be made by its manufacturer, is not guaranteed or endorsed by the publisher.

## Abbreviations

AMCF: Adult mouse cardiac fibroblast
BrdU: Bromodeoxyuridine
DR: Death receptor
KO: Knockout
LDH: Lactate dehydrogenase
LV: Left ventricular
NRVF: Neonatal rat ventricular fibroblast
OPG: Osteoprotegerin
TGF: Transforming growth factor
TPT1: Translationally controlled tumor protein 1
TRAIL: TNF-related apoptosis-inducing ligand
TUNEL: Terminal deoxynucleotidyl-transferase-mediated dUTP nick-end labeling
WGA: Wheat germ agglutinin
WT: Wild-type.
