# Supplementary material for: A Dual Role for Death Receptor 5 in Regulating Cardiac Fibroblast Function
Source: Front Cardiovasc Med. 2021 Aug 30;8:699102. doi: 10.3389/fcvm.2021.699102 (PMC8437145; doi:10.3389/fcvm.2021.699102)
Supplement: Supplementary file 1 [file Data_Sheet_1.PDF]

Supplemental Material:

Supplemental Table 1. Primer sequences used for RT-qPCR.

Gene Name	Reference Sequence	Sequence
Bax	NM_017059	Forward 5'- GTG GTT GCC CTC TTC TAC TTT -3'<br>Reverse 5'- ATC AGA ACC ATC ATG GGG CTG -3'
Bcl2	NM_016993	Forward 5'- TGG GAT GCC TTT GTG GAA CT -3'<br>Reverse 5'- GAG ACA GCC AGG AGA AAT CAA -3'
TNFRSF10B	NM_001108873	Forward 5'- ACC AGG CAG CTT TGA AGA TTA -3'<br>Reverse 5'- CTG TGC GTC CAA GAG AGA TAA A -3'
TNFRSF11B	NM_102870	Forward 5'- ACT TGG CCT CCT GCT AAT TC-3'<br>Reverse 5'- CGC ACA GGG TGA CAT CTA TT -3'
IL6	NM_012589	Forward 5'-GAA GTT AGA GTC ACA GAA GGA GTG-3'<br>Reverse 5'GTT TGC CGA GTA GAC CTC ATA G-3'
TNFA	NM_012675	Forward 3'-ACC TTA TCT ACT CCC AGG TTC T-3'<br>Reverse 3'-GGC TGA CTT TCT CCT GGT ATG-3'
TPT-1	NM_053867	Forward 5'-CTG CTG CTT ACC ATC CAT CA-3'<br>Reverse 5'-ACA ATG CCT CCA CTC CAA ATA-3'

Supplemental Table 2: Echocardiographic measurements in WT or DR5RKO BMT mice treated with vehicle or isoproterenol. Two-way ANOVA, * $p < 0.05$ versus WT Veh at same time point, † $p < 0.05$ versus WT BMT Iso at same time point. LV=left ventricle, ID=internal diameter, BPM=beats per minute, AW=anterior wall, PW=posterior wall

		LV Volume-systolic (µL)	LV Volume-diastolic (µL)	LVID-systolic (mm)	LVID-diastolic (mm)	Ejection Fraction (%)	Cardiac Output (mL/min)	Stroke Volume (µL)
**WT Veh**	Baseline	20.0±2.6	56.4±4.71	2.35±0.14	3.55±0.11	65.7±1.6	18.4±0.9	36.4±2.2
**n=10**	1 wk	22.1±2.0	58.6±4.5	2.50±0.11	3.62±0.11	62.6±0.8	18.0±1.4	36.5±2.6
**WT Iso**	Baseline	23.6±2.7	63.5±4.8	2.55±0.14	3.72±0.11	64.0±1.6	18.1±1.0	37.6±1.9
**n=12**	1 wk	35.8±3.6*	72.9±6.3*	3.0±0.13*	3.93±0.12	53.4±1.8*	19.4±1.6	39.8±3.1
**DR5KO Veh**	Baseline	21.7±1.9	58.3±3.4	2.48±0.10	3.58±0.09	63.4±1.5	17.7±1.2	36.6±1.8
**n=12**	1 wk	22.6±1.7	57.2±3.4	2.57±0.07	3.56±0.09	60.7±1.1	16.9±0.9	34.6±1.9
**DR5KO Iso**	Baseline	22.1±2.8	59.5±5.7	2.54±0.15	3.76±0.16	63.4±1.5	16.9±1.9	36.0±2.3
**n=12**	1 wk	41.0±2.5*	77.0±2.9*	3.21±0.10*	3.99±0.14	47.1±1.6*†	16.9±0.6	36.0±1.2

		Heart Rate (BPM)	LVAW-systolic (mm)	LVAW-diastolic (mm)	LVPW-systolic (mm)	LVPW-diastolic (mm)	LV Mass (mg)
**WT Veh**	Baseline	513±16	1.22±0.04	0.80±0.03	1.08±0.03	0.74±0.03	74.6±4.7
**n=10**	1 wk	512±20	1.21±0.03	0.79±0.03	1.10±0.03	0.71±0.02	76.8±4.6
**WT Iso**	Baseline	512±12	1.15±0.03	0.83±0.03	1.09±0.03	0.70±0.03	81.0±5.0
**n=12**	1 wk	493±11	1.20±0.05	0.95±0.04*	1.11±0.04	0.88±0.03*	111.8±10.2*
**DR5KO Veh**	Baseline	482±16	1.14±0.06	0.79±0.03	1.02±0.03	0.72±0.02	76.6±4.7
**n=12**	1 wk	484±14	1.05±0.03	0.75±0.02	0.93±0.04	0.66±0.03	71.3±4.3
**DR5KO Iso**	Baseline	451±20	1.16±0.05	0.77±0.02	0.98±0.05	0.70±0.05	77.8±5.6
**n=12**	1 wk	481±16	1.18±0.04	0.91±0.02*	1.02±0.04	0.84±0.03*	113.0±7.6*

Supplemental Figure 1

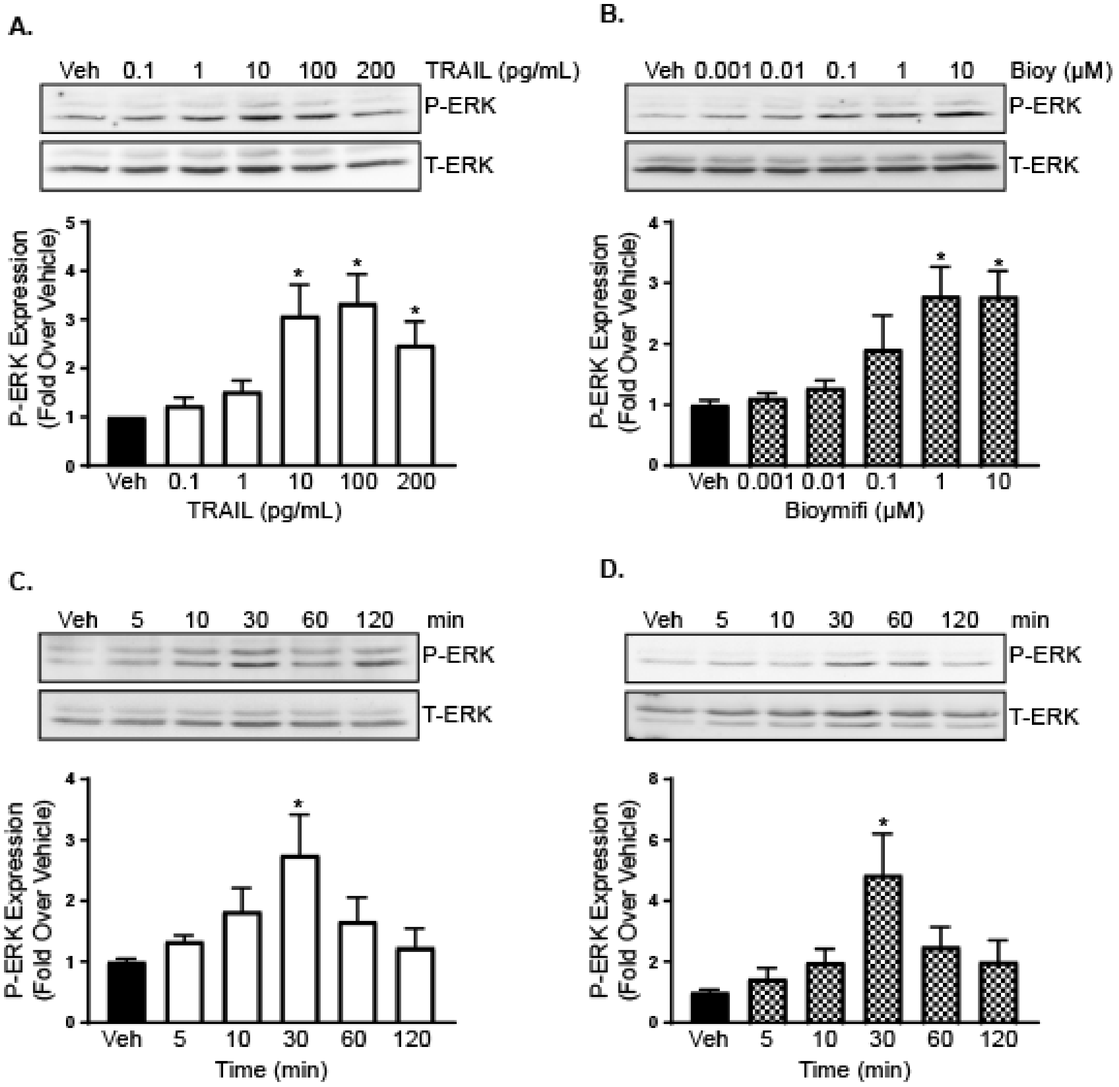

Supplemental Figure 1: NRVFs were treated with increasing doses of TRAIL (A; 0.1-200 pg/mL) or bioymifi (B; 0.001-10 μM) for 30 min and ERK1/2 activation was examined by immunoblotting for phosphorylated (P)-ERK1/2. Total (T)-ERK1/2 is shown as a loading control. P-ERK1/2 expression was normalized to T-ERK1/2 levels and expressed as fold change over vehicle treated cells. n=6, One-Way ANOVA, * $p < 0.05$ versus vehicle. The time course of DR-mediated ERK1/2 activation was determined in NRVFs treated with TRAIL (**C**) or bioymifi (**D**) over time. Lysates were immunoblotted for P-ERK1/2 and normalized to T-ERK1/2. n=6, One-Way ANOVA, * $p < 0.05$ versus vehicle.

Supplemental Figure 2

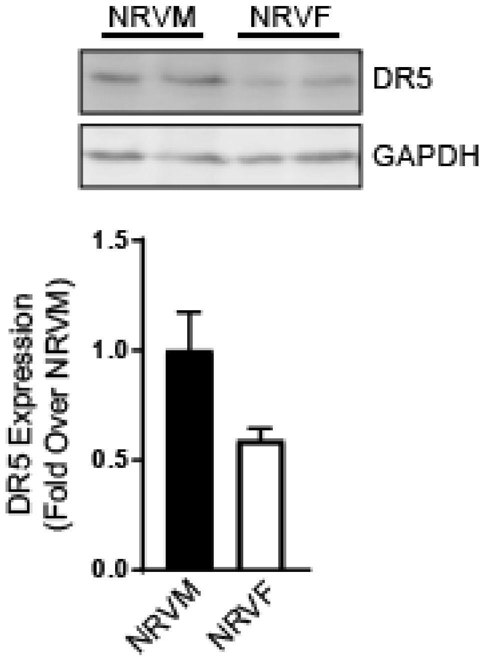

Supplemental Figure 2: Immunoblot analysis for DR5 expression in NRVMs and NRVFs. DR5 protein expression is normalized to GAPDH levels and represented as fold over NRVM levels. n=3.

Supplemental Figure 3

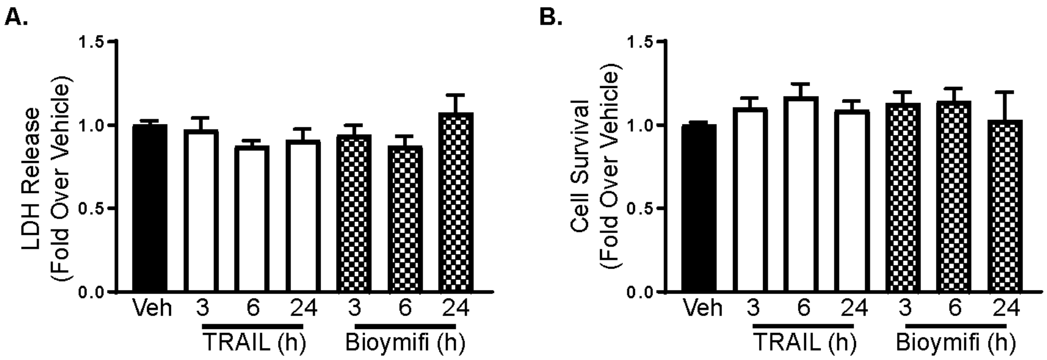

Supplemental Figure 3: A. NRVFs were treated with TRAIL or bioymifi over time and LDH release was examined in the media from cultured cells using an LDH assay. n=8. **B.** Cell viability was measured in TRAIL or bioymifi treated cells using an MTS assay. n=8.

Supplemental Figure 4

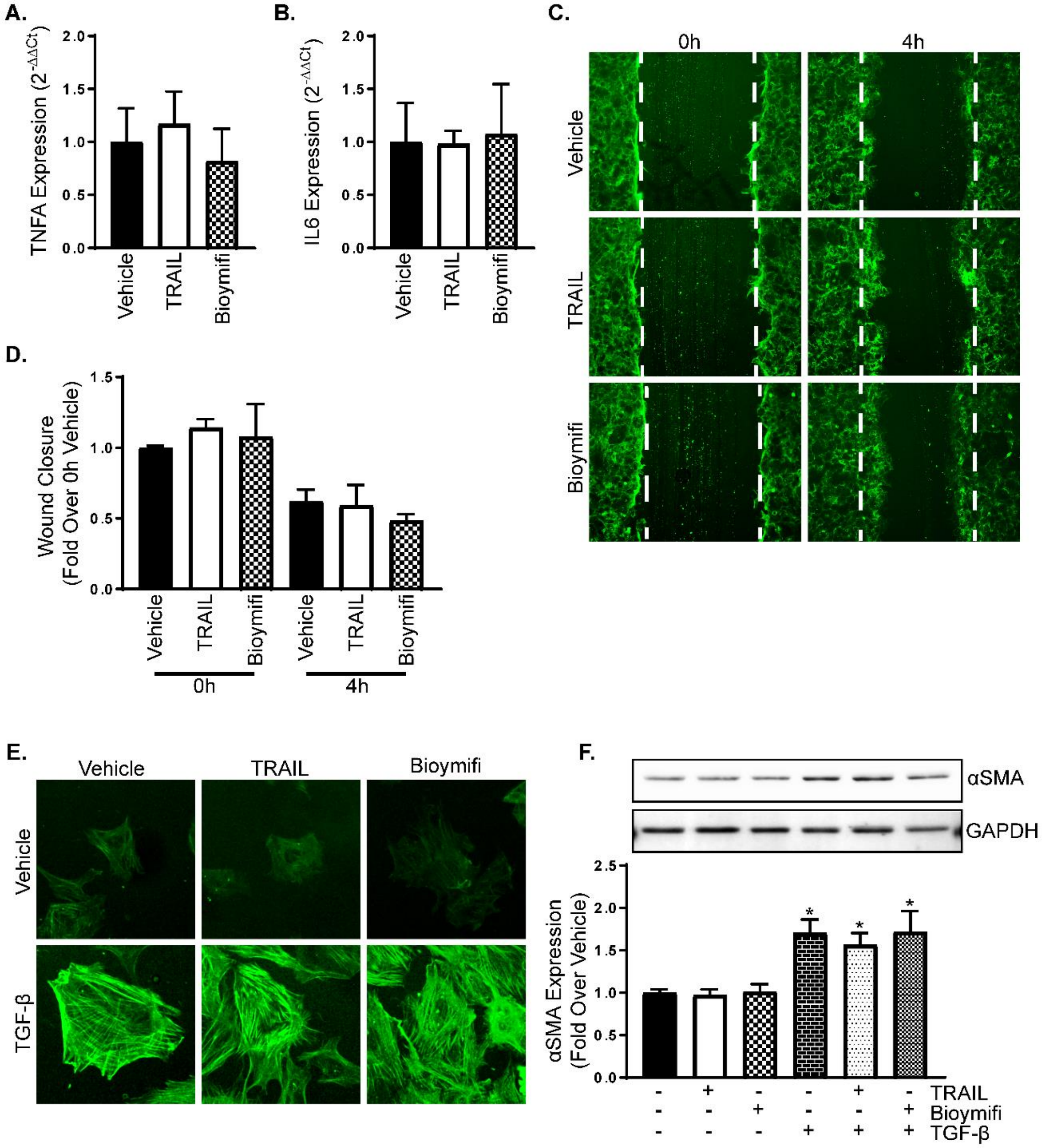

Supplemental Figure 4: Transcript expression for TNFA **(A) a**nd IL6 **(B)** was examined by RT-qPCR in NRVF treated with vehicle, TRAIL or bioymifi and expressed relative to vehicle. n=4. **C.** Representative scratch assay images **(C)** and quantified results **(D)** from NRVF at 0h and 4h post-scratch with vehicle, TRAIL or bioymifi treatment. Wheat germ agglutinin (green) was used to visualize cells. White dotted lines represent the original wound area. Values are normalized to the wound area for vehicle treated cells and time=0h. n=4. **E.** representative αSMA staining (green) for vehicle, TRAIL and bioymifi treated NRVF with or without TGF-β1 treatment. **F.** αSMA expression was quantified by immunoblot in vehicle, TRAIL and bioymifi treated NRVF with or without TGF-β1 treatment. GAPDH is shown as a loading control. Values are expressed relative to vehicle treated cells. n=6, Two-Way ANOVA, * $p < 0.05$.

Supplemental Figure 5

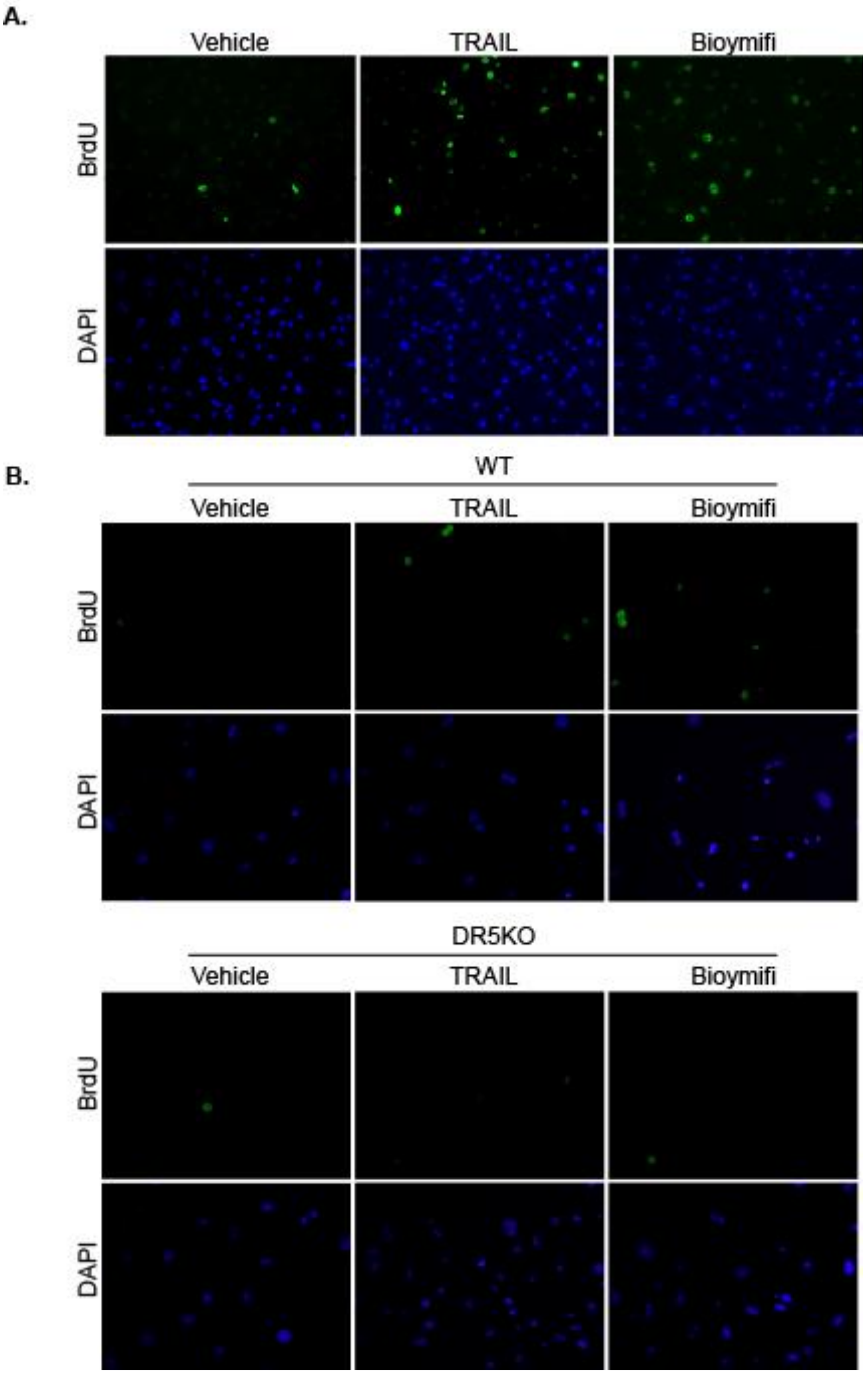

Supplemental Figure 5: A. Unmerged Brdu (green) and DAPI (blue) staining for NRVFs treated with TRAIL or bioymifi. Merged images are shown in Figure 1C. **B.** Unmerged Brdu (green) and DAPI (blue) staining for WT and DR5KO AMCFs treated with TRAIL or bioymifi. Merged images are shown in Figure 1F.

Supplemental Figure 6

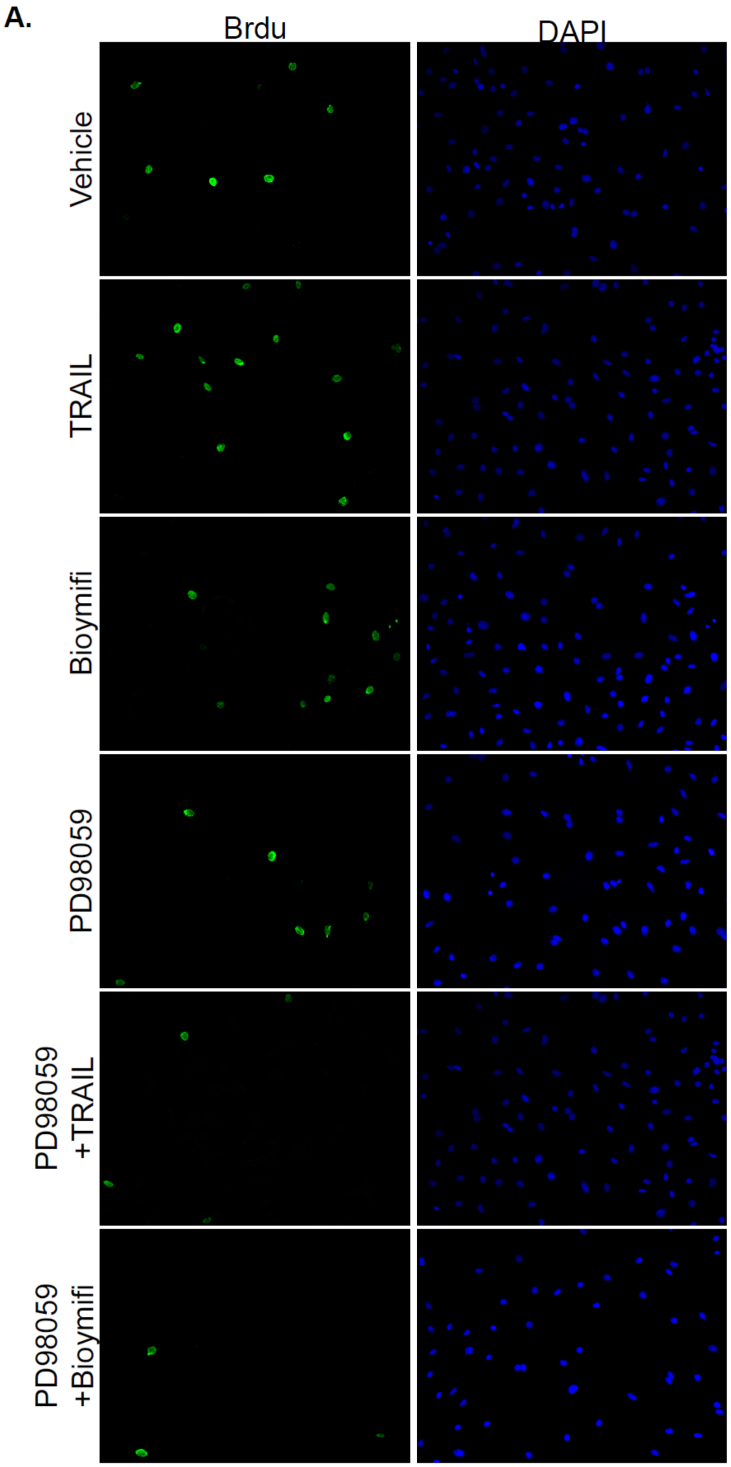

Supplemental Figure 6: A. Unmerged Brdu (green) and DAPI (blue) staining for NRVFs treated with TRAIL or bioymifi with or without PD98059 pretreatment. Merged images are shown in Figure 2C.

Supplemental Figure 7

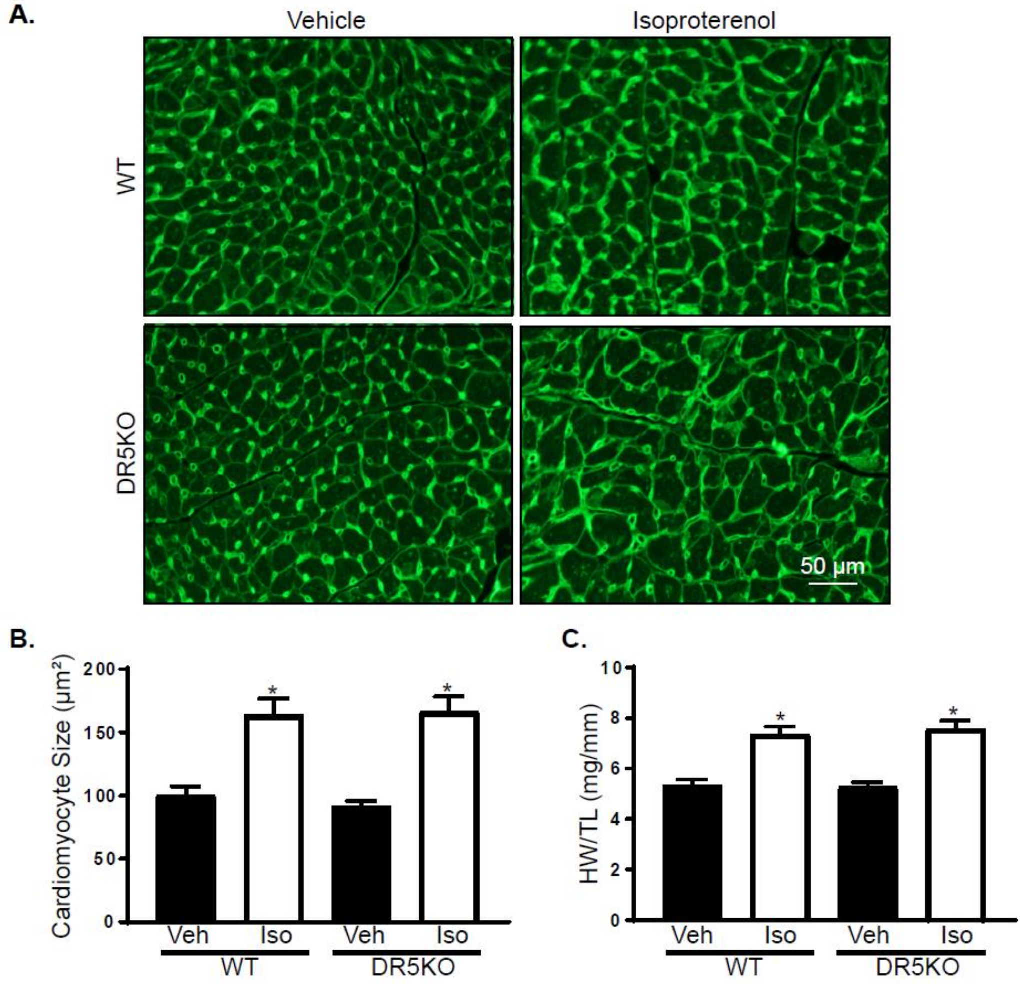

Supplemental Figure 7: Representative WGA staining **(A)** and quantification **(B)** of cardiomyocyte size for WT and DR5KO mice administered vehicle or isoproterenol. n=10 for WT vehicle, 12 for DR5KO vehicle, WT isoproterenol and DR5KO isoproterenol, Two-Way ANOVA, * $p < 0.05$ versus vehicle. **C.** Gravametric analysis of heart weight (HW) normalized to tibia length (TL) for vehicle and isoproterenol treated WT or DR5KO mice. n=10 for WT vehicle, 12 for DR5KO vehicle, WT isoproterenol and DR5KO isoproterenol, Two-Way ANOVA, * $p < 0.05$ versus vehicle.

Supplemental Figure 8

A.

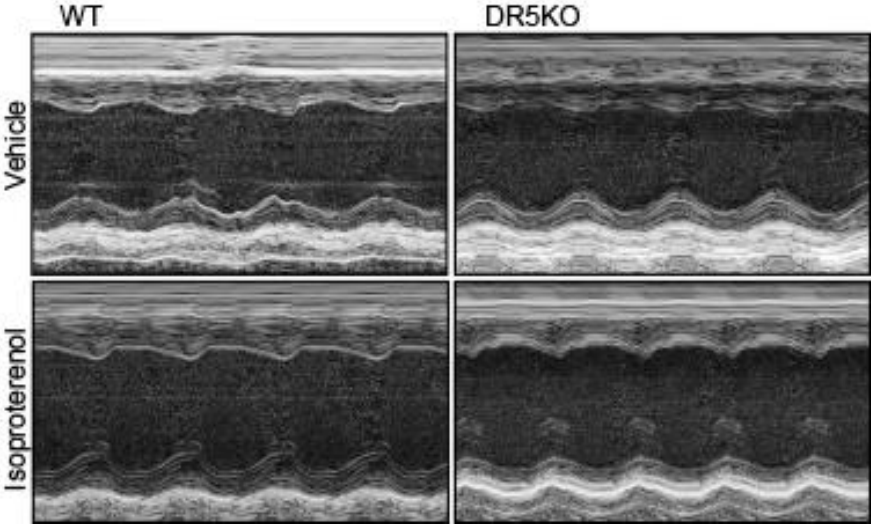

Supplemental Figure 8: Representative M-mode echocardiography images from vehicle and isoproterenol treated WT or DR5KO mice.
